# Challenges and Insights: Severe Acute Right Ventricular Dysfunction in Pulmonary Hypertension

**DOI:** 10.7759/cureus.61696

**Published:** 2024-06-04

**Authors:** Walter Y Agyeman, Julian Hawkins, Nathaniel Chishinga, Basilio Addo

**Affiliations:** 1 Internal Medicine, Piedmont Athens Regional Medical Center, Athens, USA

**Keywords:** pulmonary hypertension, right ventricle failure, ventricular function, pulmonary circulation, preventive cardiology, right ventricular failure, heart failure, right ventricle

## Abstract

Pulmonary hypertension (PH) is rarely a cause of syncope. We highlight an unusual presentation of pulmonary hypertension where management was a veritable challenge. We present a case report of a 35-year-old female with a history of stage 2 hypertension, polycystic ovarian syndrome, and obesity who presented to the hospital with a six-month history of progressive shortness of breath, lower extremity swelling, and recurrent syncope. Further evaluation with transthoracic echocardiography showed features consistent with severe pulmonary hypertension. This untreated severe pulmonary hypertension culminated in cardiogenic shock due to right ventricular (RV) failure. Successful care in this patient population entails preventing the acute downward spiral of decompensated right ventricular failure.

## Introduction

Pulmonary hypertension (PH) occurs when the mean pulmonary arterial pressure is more than 20 mmHg at rest. The definition of pulmonary arterial hypertension (PAH) also implies a pulmonary vascular resistance (PVR) of >2 Wood units and a pulmonary arterial wedge pressure of ≤15 mmHg [1]. Right ventricular (RV) failure is the terminal stage of untreated pulmonary hypertension (PH) and is a common yet underrecognized cause of morbidity and mortality. Generally, RV dysfunction portends a poor prognosis. The timely diagnosis of RV failure is of the utmost necessity due to the fundamentally different approach to management compared to other types of acute heart failure. Invariably, the mortality is high if the diagnosis is missed or delayed.

## Case presentation

A 35-year-old female with stage 2 hypertension, polycystic ovarian syndrome, and obesity presented to the hospital with a six-month history of progressively worsening dyspnea, pedal edema, fatigue, and recurrent syncope. Her symptoms acutely worsened after taking her husband's metoprolol succinate pills because her symptoms mimicked those of her husband who was taking metoprolol for heart failure with reduced ejection fraction. Before this, she worked in a podiatry office, running errands and caring for her three children without any respiratory limitations. Syncopal episodes were provoked by exertion, and this progressed to syncope while standing at rest. She denied chest pain, snoring, birth control medications, or personal history of miscarriages. She had no history of pulmonary hypertension or connective tissue disease. She had preeclampsia in two previous pregnancies. Family history was significant for recurrent pulmonary embolism, coronary artery disease, femoral pseudoaneurysm repair, and rheumatoid arthritis in her father.

Due to her symptoms, she established care with her primary care doctor two months prior. Her primary care doctor had prescribed furosemide, losartan, and hydrochlorothiazide and ordered an echocardiogram. The echocardiogram was not done due to financial issues. She was a former tobacco smoker but denied alcohol, amphetamine, or other substance use. She had been taking phentermine for two years, losing about 100 lbs. She had pitting edema of her legs and peripheral cyanosis. She was hypotensive with positive orthostatic vitals. There were no crackles or wheezing on auscultating her lungs. She had an elevated brain natriuretic peptide (BNP) of 469.9, elevated lactate of 3.5, acute kidney injury, and urine drug screen positive for amphetamines. Her total bilirubin was 2.9, and prothrombin time (PT)/international normalized ratio (INR) was 46.5 seconds/4.01. Her white cell count was 15.9 × 10^3^/µL. Her complete blood count values are summarized in Table 1. She met sepsis criteria with leukocytosis and fever with the focus of infection suspected to be a urinary tract infection, making management even more challenging. She was initially fluid-resuscitated and received empiric antibiotic therapy.

**Table 1:** Results of Serology and Chemopathology Tests CO_2_, carbon dioxide; BUN, blood urea nitrogen; GFR, glomerular filtration rate; CKD-EPI, Chronic Kidney Disease Epidemiology Collaboration

Basic metabolic panel	Values	Reference range and units
Sodium	140	133-145 mmol/L
Potassium	3.4	3.3-5.1 mmol/L
Chloride	105	98-108 mmol/L
CO_2_	23	22-32 mmol/L
Glucose	78	70-100 mg/dL
BUN	30	6-20 mg/dL
Creatinine	1.29	0.40-1.00 mg/dL
Calcium	9.5	8.4-10.2 mg/dL
Anion gap	12	8-12
BUN/creatinine ratio	23	12-20
GFR CKD-EPI	56	≥60 mL/minute/1.73 m^2^
Hepatic function panel		
Total protein	6.1	5.9-8.4 g/dL
Albumin	3.4	3.0-5.0 g/dL
Alanine aminotransferase	42	7-52 U/L
Aspartate aminotransferase	47	12-42 U/L
Alkaline phosphatase	67	32-126 U/L
Total bilirubin	1.2	0.2-1.4 mg/dL
Bilirubin, direct	0.42	0.00-0.30 mg/dL
Bilirubin, indirect	0.8	0.1-1.1 mg/dL
Albumin/globulin ratio	1.3	1.1-2.2
Other serology results		
Troponin	16	<16 ng/L
Brain natriuretic peptide	469.9	10-100 pg/mL
Urine pregnancy test	Negative, <0.6	
Prothrombin time	17.1	9.4-12.5 seconds
International normalized ratio	1.52	0.9-1.2
C-reactive pain	0.6	0.02-1 mg/dL
Blood culture	No growth after five days	
Lactate	3.5	0.5-2.00 mmol/L
Ferritin	15.5	11-306.80 ng/mL
Iron	25	30-160 μg/dL
Total iron-binding capacity	416	228-428 μg/dL
Iron saturation	6	20%-55%
Cortisol morning level	34.1	6.7-22.6 μg/dL

**Figure 1:**
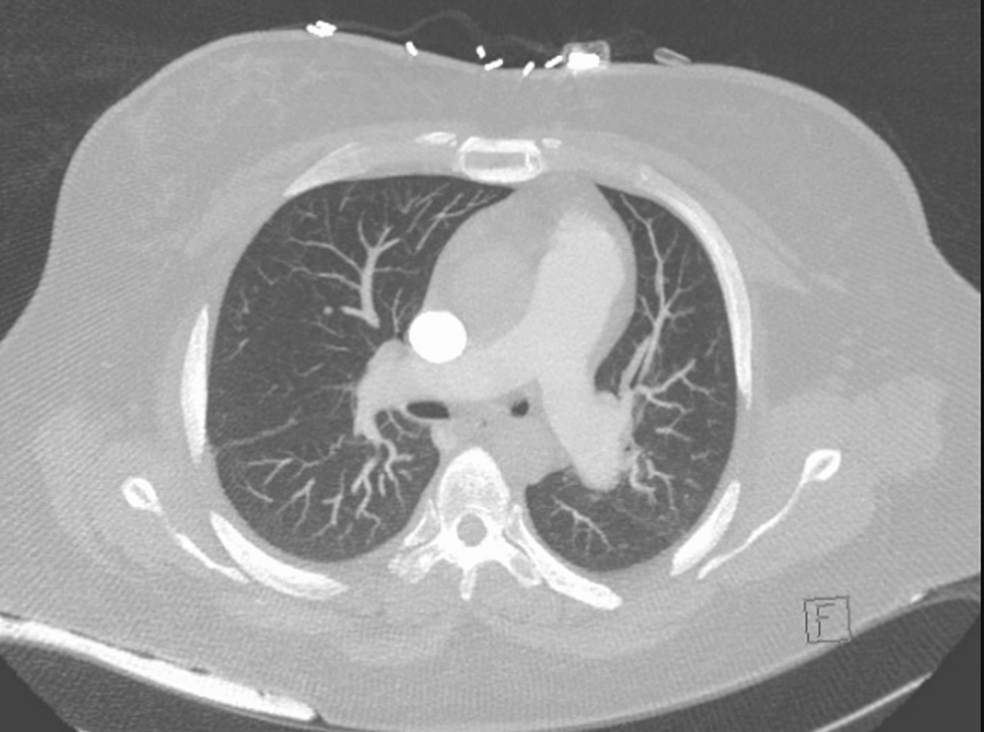
Computed Tomography Angiogram of the Chest

A stat chest computed tomography (CT) angiogram with contrast was negative for pulmonary embolism and showed small bilateral pleural effusions and lower lobe atelectasis. The CT angiogram of the chest showed reflux of contrast into the hepatic veins and intrahepatic inferior vena cava, consistent with elevated right heart pressures (Figure 1).

**Figure 2:**
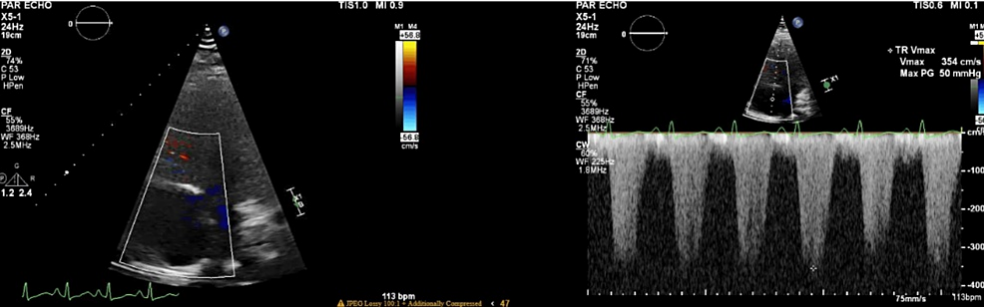
Transthoracic Echocardiogram Showing Tricuspid Regurgitant Max Jet Velocity (TR Vmax)

**Figure 3:**
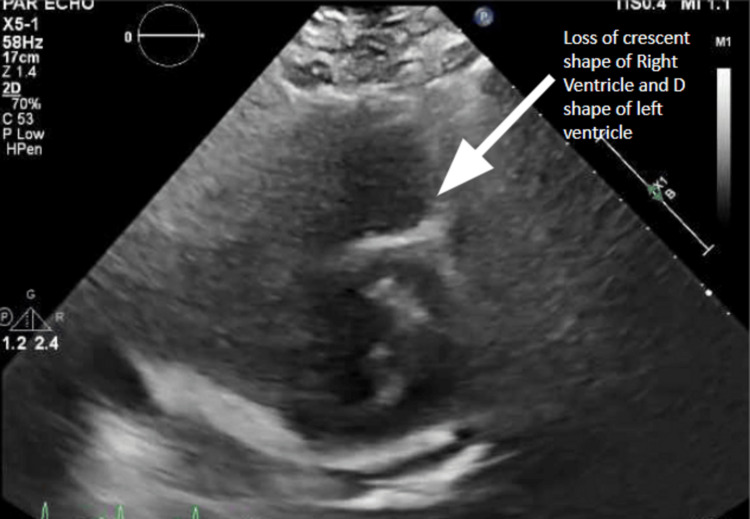
Transthoracic Echocardiogram Showing Parasternal Short Axis View With Loss of Normal Crescent Shape of Right Ventricle and D "Sign" (Cross Section of Left Ventricle Shaped Like the Letter "D")

**Figure 4:**
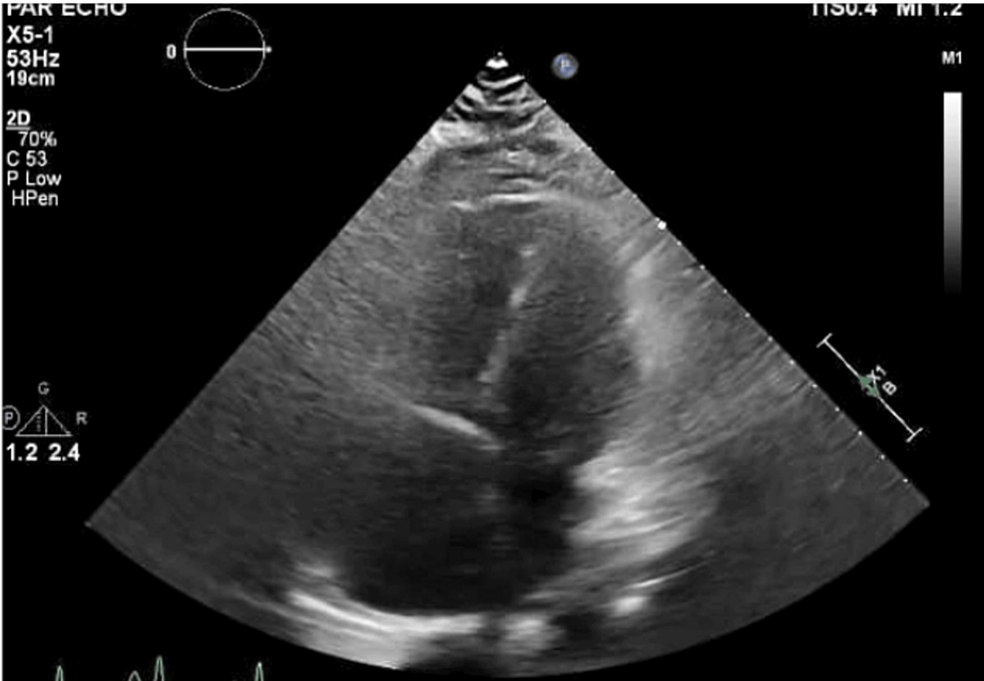
Transthoracic Echocardiogram Showing Apical Four-Chamber View With Dilated Right Ventricle and Right Atrium

A transthoracic echocardiogram revealed an ejection fraction of 55%, concentric left ventricular (LV) hypertrophy, severe RV dilation, diastolic septal flattening, and severely elevated RV systolic pressure of 72 mmHg. Notably, the tricuspid annular plane systolic excursion (TAPSE) was 1.4 mm. Her TAPSE was consistent with a mild degree of right ventricular dysfunction. She had reduced RV function and moderate tricuspid regurgitation (Figures 2-4).

She had positive echocardiographic evidence of untreated pulmonary hypertension leading to cardiogenic shock due to RV failure. This is supported by elevated BNP, tachycardia despite fluids, peripheral ischemia, and elevated lactate. A serologic workup including antinuclear antibody (ANA), anti-cyclic citrullinated peptide (CCP) antibodies, and HIV screen was negative (Table 2).

**Table 2:** Laboratory Results of Complete Blood Count

Complete blood count	Values	Reference range and units
White cell count	15.9	4-12 × 10^3^/µL
Red blood cell count	6.06	3.7-5/0 × 10^6^/µL
Hemoglobin	12.6	12-16 g/dL
Mean cell volume	67.6	78-100 fL
Mean cell hemoglobin	20.8	27-34 pg
Mean cell hemoglobin concentration	30.7	32-36 g/dL
Red cell distribution width	20.9	11.6%-14.5%
Mean platelet volume	7.3	7.5-11.2 fL
Platelets	347	130-400 × 10^3^/µL
Neutrophil, relative	67.7	40%-75%
Lymphocytes, relative	23	18%-48%
Monocytes, relative	8	0%-12%
Eosinophils, relative	0.4	0%-6%
Basophils, relative	0.9	0%-2%

The patient was transferred to the intensive care unit due to cardiogenic shock. She was intubated and initially received a dobutamine drip, but due to tachycardia, she was transitioned to milrinone and norepinephrine infusions. A Swan-Ganz catheter was placed to monitor pulmonary hemodynamics, as shown in Table 3. She was initiated on intravenous (IV) treprostinil, as well as inhaled nitric oxide. Remodulin was titrated with close monitoring of her mixed venous gas. Her initial mixed venous gas before starting milrinone was 43% from the pulmonary arterial catheter and went up to 70%. Due to severe metabolic acidosis, she received continuous renal replacement therapy with a bicarb bath. She developed acute liver injury with associated coagulopathy secondary to congestive hepatopathy due to cardiogenic shock and RV failure. As a result of her multiorgan failure, she was not a candidate for venoarterial extracorporeal membrane oxygenation. She developed cardiac arrest and was unsuccessfully resuscitated.

**Table 3:** Hemodynamic Parameters Obtained With Emergency Right Heart Catheterization WU: Wood units

Hemodynamic characteristics	Values	Measured variables: Normal value
Right atrial pressure	18 mmHg	2-6 mmHg
Right ventricular pressure	77/20 mmHg	25/5 mmHg
Pulmonary arterial pressures	97/40 mmHg	Pulmonary artery pressure, systolic (sPAP): 15-30 mmHg; pulmonary artery pressure, diastolic (dPAP): 4-12 mmHg
Pulmonary artery (PA) pressure, mean (mPAP)	59 mmHg	8-20 mmHg
Pulmonary capillary wedge pressure (PCWP), mean	Not obtained	≤15 mmHg
Mixed venous oxygen saturation (SvO_2_)	43% from the PA catheter	65%-80%
Arterial oxygen saturation (SaO_2_)	95% on 15 L	95%-100%
CO/CI	2.6 L per minute/1.2 L per minute per meter squared	Cardiac index (CI), 2.5-4.0 L/minute·m^2^; cardiac output (CO), 4-8 L/minute
Pulmonary vascular resistance	Not obtained	Pulmonary vascular resistance (PVR): 0.3-2.0 WU

## Discussion

Pulmonary hypertension (PH) is prevalent in the general population and associated with increased mortality. The diagnosis and treatment of pulmonary hypertension can be quite challenging. Appropriate management requires understanding the classification, risk factors, and clinical features of pulmonary hypertension. Pulmonary hypertension (PH) refers to a mean pulmonary arterial pressure of >20 mmHg at rest. The precapillary subtype of PH is defined with a pulmonary vascular resistance (PVR) of >2 Wood units and a pulmonary arterial wedge pressure of ≤15 mmHg [1]. Other subtypes of PH are defined in Table 4. The early symptoms of pulmonary hypertension include dyspnea with exertion, fatigue, exercise intolerance, bendopnea, cough, palpitations, hemoptysis, and syncope. Rarely, patients may experience exertional chest pain due to the dynamic compression of the left main coronary artery or hoarseness (Ortner's syndrome) [1]. The risk factors include anorexigenic drugs, amphetamines, chemotherapeutic drugs, and dasatinib.

**Table 4:** Hemodynamic Classification of Pulmonary Hypertension mPAP, mean pulmonary arterial pressure; PAWP, pulmonary arterial wedge pressure; PVR, pulmonary vascular resistance; WU, wood units; CO, cardiac output

Definition	Hemodynamic characteristics
Pulmonary hypertension	mPAP of >20 mmHg
Precapillary pulmonary hypertension	mPAP of >20 mmHg, PAWP of ≤15 mmHg, and PVR of >2 WU
Isolated postcapillary pulmonary hypertension	mPAP of >20 mmHg, PAWP of >15 mmHg, and PVR of ≤2 WU
Combined pre- and post-pulmonary hypertension	mPAP of >20 mmHg, PAWP of >15 mmHg, and PVR of >2 WU
Exercise pulmonary hypertension	mPAP/CO slope between rest and exercise of >3 mmHg/L/minute
Unclassified pulmonary hypertension	mPAP of >20 mmHg, low PVR of ≤2 WU, and low PAWP of ≤15 mmHg

The patient's symptoms of syncope and worsening kidney function occurred due to chronic untreated pulmonary hypertension, culminating in hypotension from severe RV failure. She presented acutely after taking her husband's oral metoprolol for her dyspnea. Her positive rheumatoid factor, low C3 and low C4 complement levels, and elevated creatinine kinase suggest that she may have had mixed connective tissue disease that presented as isolated pulmonary hypertension. The etiology of her PAH is probably idiopathic, connective tissue disease-related given her family history of rheumatoid arthritis, or related to phentermine (Adipex). The US FDA approved phentermine for treating obesity in 1959. Phentermine is structurally similar to other anorexigenic drugs, including fenfluramine, aminorex, and amphetamines, which have a definite association with PH (Figure 1). Fenfluramine has been banned since November 1997 by the US FDA due to its significant association with PH [2,3]. Although there is a case report of phentermine as a possible cause of PH, there have been no other studies, controlled or otherwise, corroborating this [2,4]. However, it has been disputed that phentermine is associated with PH [4].

The patient presented with sepsis with leukocytosis and fever, making management even more challenging. She was initially fluid-resuscitated, received empiric antibiotic therapy, and had an appropriate improvement in her blood pressure. However, fluid therapy triggered her right ventricle's spiraling dysfunction due to her preexisting pulmonary hypertension. The right ventricular death spiral occurs from hemodynamic collapse from impaired LV filling and the hypoperfusion of the RV myocardium secondary to RV dilatation. Unlike the left ventricle, the RV is perfused during systole and diastole. As the pulmonary artery systolic pressure approaches the systolic pressure, perfusion is impaired during diastole in RV failure [5]. Interventions that increase the systolic blood pressure and decrease the pulmonary artery systolic pressure are required to maintain perfusion [6,7]. Excessive RV preload shifts the interventricular septum, causing diastolic compression of the left ventricle and reduced cardiac output. Patients with severe right ventricular dilatation, including those with acute pulmonary embolism, derive little benefit from volume support and may improve with diuresis [7,8].

Significant pulmonary hypertension can be noted on a CT scan as demonstrated by an RV/LV diameter ratio of >1 and a pulmonary artery dilation/aorta ratio of >1. Contrast reflux into the inferior vena cava and hepatic veins suggests right ventricular insufficiency. RV failure is clearly appreciated in the subcostal four-chamber view on the transthoracic echocardiogram. In moderate RV dilation, the RV is 60%-100% the size of the LV. In severe RV dilation, the RV is larger than the LV. The D sign refers to septal flattening observed in short axis views of the heart (Figure 2). Tricuspid annular plane systolic excursion (TAPSE) is an excellent indicator of RV systolic function at the bedside assessed via M-mode from the apical four-chamber view. TAPSE is graded as normal TAPSE, >17 mm; mild RV dysfunction, 10-17 mm; moderate RV dysfunction, 5-10 mm; and severe RV dysfunction, <5 mm. In our patient, she had a mild degree of right ventricular dysfunction as determined by her TAPSE value of 14 mm. However, she descended into right ventricular failure as she had acute right heart strain. Acute right heart strain is a distinct echocardiographic feature that can be seen using McConnell's sign, a regional pattern of RV dysfunction, with akinesia of the mid-free wall and hypercontractility of the apical wall.

To avoid adverse outcomes in patients with PH who present with RV failure, physicians must have a high index of suspicion for PH in patients with dyspnea and syncope. In such situations, an early mixed venous oxygen saturation and a transthoracic echo are required to help guide emergent management and truncate the right ventricular spiral of death. Before starting pulmonary vasodilator therapy, invasive hemodynamic testing should be employed [9]. In those patients with pulmonary hypertension and pulmonary vascular resistance, inhaled nitric oxide provides clinical benefit by causing pulmonary vascular dilation and improving ventilation-perfusion mismatch. Prostacyclins are indicated for patients in WHO functional class 3-4 heart failure; treprostinil is indicated explicitly for WHO class 3, especially in group 1 pulmonary arterial hypertension [1].

## Conclusions

Pulmonary hypertension requires a high index of suspicion and can present idiopathically as recurrent syncope. It may be detected in adult patients in their fourth decade of life with dyspnea and recurrent syncope as primary symptoms. The simplified diagnostic algorithm for pulmonary hypertension includes a three-step process from physician suspicion, echocardiography detection, and confirmation with right heart catheterization in pulmonary hypertension centers. Early detection is essential for subsequent appropriate medical or surgical intervention to prevent mortality.
